# The expression and prognostic value of protein tyrosine kinase 6 in early-stage cervical squamous cell cancer

**DOI:** 10.1186/s40880-016-0114-2

**Published:** 2016-06-16

**Authors:** Xiao-Jing Wang, Ying Xiong, Ze-Biao Ma, Jian-Chuan Xia, Yan-Fang Li

**Affiliations:** Sun Yat-Sen University Cancer Center, State Key Laboratory of Oncology in South China, Collaborative Innovation Center of Cancer Medicine, Guangzhou, 510060 Guangdong P. R. China; Department of Gynecologic Oncology, Sun Yat-Sen University Cancer Center, Guangzhou, 510060 Guangdong P. R. China; Department of Biotherapy, Sun Yat-Sen University Cancer Center, Guangzhou, 510060 Guangdong P. R. China

**Keywords:** Cervical squamous cell cancer, PTK6, Prognosis, Immunohistochemistry

## Abstract

**Background:**

Protein tyrosine kinase 6 (PTK6) is overexpressed in many epithelial tumors and predicts poor prognosis. However, PTK6 expression status and its role in cervical squamous cell cancer are unknown. This study aimed to investigate the expression level and clinical significance of PTK6 in early-stage cervical squamous cell cancer.

**Methods:**

Quantitative reverse transcription-polymerase chain reaction (qRT-PCR) and western blotting analysis were performed to detect PTK6 mRNA and protein expression levels in 10 freshly frozen, early-stage cervical squamous cell cancer specimens and adjacent non-tumorous cervical tissues. The expression of PTK6 was detected using immunohistochemical staining in 150 formalin-fixed, paraffin-embedded, early-stage cervical squamous cell cancer sections and 10 normal cervical tissue sections.

**Results:**

The mRNA and protein levels of PTK6 in cancer tissues were higher than those in adjacent non-tumorous cervical tissues. Immunohistochemical analysis showed that PTK6 was not expressed in normal cervical tissues but was overexpressed in the cytoplasm of cervical squamous cell cancer cells. The level of PTK6 expression was significantly associated with tumor grade (*P* = 0.020). The 5-year overall survival rate of patients with high PTK6 expression was lower than that of patients with low PTK6 expression (81.3% vs. 96.2%, *P* = 0.008). Multivariate Cox regression analysis showed that the expression level of PTK6 in cervical squamous cell cancer was an independent prognostic factor for patient survival (hazard ratio = 5.999, 95% confidence interval 1.622–22.191, *P* < 0.05).

**Conclusions:**

PTK6 is overexpressed in cervical squamous cell cancer. Increased PTK6 expression is associated with reduced 5-year overall survival. PTK6 expression is an independent prognostic predictor for cervical cancer.

## Background

Cervical cancer is the most common malignancy of the female genital tract worldwide and the second leading cause of cancer death in women. Approximately 275,000 deaths due to cervical cancer occurred worldwide in 2008, and approximately 85% of these cases occurred in developing countries [1]. The substantial decline of its incidence and mortality in developed countries was presumed to reflect the use of effective screening and anti-human papillomavirus (HPV) vaccines [2]. In 2011, there were an estimated 87,982 new cases in China, and the annual number of deaths was greater than 20,000 [3]. Approximately 75% of all cervical cancer patients have squamous cell carcinoma [4]; however, the incidence of cervical adenocarcinoma has increased in the past three decades [5]. The main treatment of early-stage disease is either a radical hysterectomy and pelvic lymph node dissection or radiotherapy; the main treatment of late-stage disease is radiotherapy. Despite the improvement of surgical skill and radiotherapy methods, the 5-year survival rate for patients with late-stage disease remains unsatisfactory. Therefore, understanding the molecular mechanisms of the early events of cervical cancer and searching for novel targets involved in the progression of cervical cancer is of great value for providing new therapeutic targets and improving patient survival.

Protein tyrosine kinase 6 (PTK6), also termed breast tumor kinase (Brk), was initially identified in a metastatic breast tumor [6], subsequently cloned from mouse intestinal crypt cells and named Src-related intestinal kinase (Sik) [7]. PTK6 protein structure consists of the Src homology 3 (SH3) domain, Src homology 2 (SH2) domain, and catalytic domains, which structurally resemble the Src family of non-receptor protein tyrosine kinases. The most notable distinction between PTK6 and the SRC family kinases is the lack of myristoylation and palmitoylation sequences, which make intracellular localization flexible. PTK6 has been implicated in a variety of tissues and cancers and is related to the regulation of different signaling pathways. A growing number of studies have revealed that the function of PTK6 depends on the cell type it is expressed in and its intracellular localization. PTK6 is overexpressed in many epithelial tumors, including breast cancer [8], non-small cell lung cancer [9], and ovarian cancer [10], and is associated with poor patient survival. However, in other tumors, including nasopharyngeal carcinoma [11] and esophageal squamous cell carcinoma [12], PTK6 may play an important anti-oncogenic role by regulating cell proliferation, differentiation, and migration. The role that PTK6 plays in different tumors reveals its tissue specificity. Compared with the frequency of ErbB2 overexpression (25%–30%), the high frequency of PTK6 expression (60%–86%) in breast tumor cells suggests that this protein has great potential as a new therapeutic target, especially in patients with no ErbB2 expression. The expression level of PTK6 and its clinical relevance in cervical squamous cell cancer have not been studied. In this study, we investigated the expression level and clinical significance of PTK6 in early-stage cervical squamous cell cancer.

## Methods

### Patients and tissue specimens

The inclusion criteria for patients were as follows: (1) a pathologically confirmed diagnosis of cervical squamous cell cancer and an International Federation of Obstetrics and Gynecology (FIGO) stage between IB1 and IIB; (2) initial treatment including radical hysterectomy and bilateral pelvic lymph node dissection were performed at Sun Yat-sen University Cancer Center between January 2009 and December 2011; (3) no preoperative anti-cancer therapy; and (4) no secondary cancer. Paraffin-embedded cervical squamous cell cancer specimens were obtained from the Pathology Department. Normal cervical epithelial samples (to be used as normal controls) were collected from patients who had benign uterine tumors and needed a hysterectomy during the same period. Patients’ hospital records were reviewed to obtain demographic data, including age, serum level of squamous cell carcinoma (SCC) antigen, tumor size and stage, surgical procedures, pathologic report, adjuvant therapy, and follow-up information.

Postoperative adjuvant radiotherapy was recommended for patients with the following pathologic risk factors: positive lymph nodes, deep cervical stromal invasion, positive margin, and/or lymphovascular space invasion. Postoperative chemotherapy was recommended for patients with positive lymph nodes or lymphovascular space invasion. The sequential chemotherapy regimens were as follows: irinotecan at 60–80 mg/m^2^ on days 1 and 8 or paclitaxel at 175 mg/m^2^ and cisplatin at 60–75 mg/m^2^ on day 1. These doses were repeated every 3 weeks for 4 cycles. The concurrent chemotherapy regimen was cisplatin at a dose of 35–40 mg/m^2^ weekly during radiotherapy. The total radiation dose was approximately 45–50 Gy with a fractionation of 1.8–2.0 Gy daily. The irradiation fields were mainly the pelvic cavity field with two exceptions of a para-aortic extended field.

Ten fresh cervical squamous cell cancer specimens (from 6 patients with FIGO stage IB1 tumor and 4 patients with FIGO stage IIA1 tumor; 2, 3, and 5 of the 10 patients had grade 3, 2, and 1 tumors, respectively) and their matched adjacent non-tumorous cervical tissues were collected immediately after resection and were immersed in RNAlater (Ambion, Austin, TX, USA) to prevent RNA degradation. The samples were stored at 4 °C overnight and then frozen at −80 °C before RNA and protein extraction for quantitative reverse transcription polymerase chain reaction (qRT-PCR) and Western blotting analysis.

### qRT-PCR

Total RNA was extracted from frozen tissues using Trizol reagent (Invitrogen, Carlsbad, CA, USA) in accordance with the manufacturer’s instructions. A Nanodrop Spectrophotometer (ND-1000; Thermo Scientific, Wilmington, DE, USA) was used to assess the concentration and quality of the extracted total RNA. According to the manufacturer’s instruction, cDNA was synthesized using 2 μg RNA and M-MLV Reverse Transcriptase (Promega, Fitchburg, WI, USA). We used the ABI 7900HT Real-time PCR system (Life Technologies, Carlsbad, CA, USA) to perform gene amplification with the following reaction conditions: 94 °C for 5 min; 40 cycles at 94 °C for 30 s, 58 °C for 30 s, and 72 °C for 50 s for primer extension; and 72 °C for 10 min. The housekeeping gene glyceraldehyde-3-phosphate dehydrogenase (GAPDH) was used as an internal control. The primers for qRT-PCR were designed using the primer 5.0 software (PREMIER Biosoft International, Palo Alto, CA, USA) and had the following sequences: 5′-TACTTTGGGGAGGTCTTCGAG-3′ for forward PTK6 primer and 5′-TGCCGCAGCTTCTTCATG-3′ for reverse PTK6 primer; 5′-CTCCTCCTGTTCGACAGTCAGC-3′ for forward GAPDH primer and 5′-CCCAATACGACCAAATCCGTT-3′ for reverse GAPDH primer. The comparative cycle threshold (Ct) value was measured and used for data analysis.

### Western blotting analysis

Total protein was extracted from frozen samples using Radio-Immunoprecipitation Assay Lysis Buffer, and the total protein concentration was measured using the Bicinchoninic Acid Protein Assay Kit (Bio-Rad, Hercules, CA, USA). Proteins were separated by sodium dodecyl sulfate–polyacrylamide gel electrophoresis (SDS-PAGE) and then electro-transferred onto a polyvinylidene difluoride (PVDF) membrane (Bio-Rad). The blotted membranes were blocked in 5% skim milk diluted in phosphate buffer solution supplemented with 0.1% Tween (PBST) for 1 h. After blocking the macromolecular antigen, the blotted membranes were incubated with anti-rabbit PTK6 antibody (1:1000 dilution; Proteintech, Wuhan, Hubei, China) followed by horseradish peroxidase (HRP)-conjugated secondary antibody (1:2000 dilution; Cell Signaling Technologies, Danvers, MA, USA) for 1 h. GAPDH protein (1:2000 dilution; Proteintech) was used as a loading control.

### Immunohistochemistry

Paraffin-embedded cervical squamous cell cancer specimens were cut into 5-mm thick sections and then dewaxed with xylene. After rehydration in gradient alcohol solutions, these sections were submerged in ethylene diamine tetraacetic acid (EDTA) buffer and microwaved in a cooker at high pressure for 12 min followed by low pressure for 13 min for antigen retrieval. To quench endogenous peroxidase and non-specific binding, these sections were incubated in 3% hydrogen peroxide for 10 min followed by submergence in fetal bovine serum for 30 min at room temperature. The tissue sections were incubated with anti-PTK6 antibody (1:500 dilution) at 4 °C overnight followed by HRP-conjugated secondary antibody after washing with phosphate-buffered saline (PBS) five times (5 min each time). Subsequently, the visualization signal was developed with diaminobenzidine tetrahydrochloride (DAB) for 1 min and counterstained with hematoxylin.

The results of immunohistochemical (IHC) staining were scored independently by two pathologists regardless of patients’ clinical features. The IHC score was determined by both staining intensity and proportion of positively stained cancer cells. Intensity of staining score was categorized as 0, no staining; 1, weak staining; 2, moderate staining; and 3, strong staining. The percentage of stained tumor cell was scored as 0, <10%; 1, ≥10% to <25%; 2, ≥25% to <50%; 3, ≥50% to <75%; and 4, ≥75%. The final immunoreactivity score (IRS) was the product of staining intensity score and percentage score and ranged from 0 to 12. Cut-off values for PTK6 expression were based on the median of all products. An optimal cut-off value was determined as follows: ≤6 indicates low PTK6 expression, and >6 indicates high PTK6 expression.

### Statistical analysis

Statistical Product and Service Solutions (SPSS) software package (version 17.0, SPSS Inc., Chicago, IL, USA) was used to perform statistical analysis. The Chi square test or Fisher’s exact test was used to determine the relationship between PTK6 expression levels and the clinicopathologic features of patients with cervical squamous cell cancer. Patients were followed up by either outpatient visit or telephone survey before January 31, 2015, and recurrence was diagnosed based on clinical and laboratory assessments. The overall survival (OS) of patients was defined as the time from initial surgery until the date of death or the last follow-up. The progression-free survival (PFS) of patients was determined as the time from initial surgery to recurrence or progression. Kaplan–Meier analysis was employed to plot survival curves, and the log-rank test was used to compare the differences between the survival curves. The prognostic effects of clinicopathologic variables were identified by univariate and multivariate Cox proportional hazards regression analysis. A *P* < 0.05 was considered statistically significant.

## Results

### Clinical features of included patients with early-stage cervical squamous cell cancer

A total of 150 patients were selected from our cancer center database between January 2006 and December 2011. The median follow-up duration was 57 months (range, 17–100 months). The median patient age was 45 years (range, 31–67 years). The numbers of patients with stage IB1, IB2, IIA1, and IIB disease were 99, 12, 34, and 5, respectively. 6, 38, and 106 patients had grade 1, 2, and 3 tumors, respectively. The metastasis rate of the pelvic lymph nodes was 27.3% (41/150) (Table 1). Eighty-one patients received postoperative pelvic radiotherapy. Of the 81 patients, 49 received radiotherapy plus cisplatin-based chemotherapy (28 received sequential chemoradiotherapy, and 21 received concurrent chemoradiotherapy), and 32 received radiotherapy alone. Of the total 150 patients, 4 received chemotherapy alone as postoperative adjuvant therapy.

**Table 1:** Relationships between protein tyrosine kinase 6 (PTK6) expression level and clinicopathologic characteristics of the patients with early-stage squamous cervical cell cancer

Characteristic	Total (cases)	PTK6 expression [cases (%)]		χ^2^ value	*P* value
		High	Low		
Age (years)				0.172	0.679
≤45	81	36 (44.4)	45 (55.6)		
>45	69	33 (47.8)	36 (52.2)		
Tumor size (cm)				0.842	0.359
≤4	138	65 (47.1)	73 (52.9)		
>4	12	4 (33.3)	8 (66.7)		
Tumor grade				7.651	0.020*
1	6	6 (100)	0 (0)		
2	38	18 (47.4)	20 (52.6)		
3	106	45 (42.5)	61 (57.5)		
Clinical stage				3.484	0.323
IB1	99	45 (45.5)	54 (54.5)		
IB2	12	4 (33.3)	8 (66.7)		
IIA1	34	19 (55.9)	15 (44.1)		
IIB	5	1 (20.0)	4 (80.0)		
Pelvic lymph node metastasis				0.176	0.675
Absent	109	49 (45.0)	60 (55.0)		
Present	41	20 (48.8)	21 (51.2)		
Deep cervical stromal invasion				2.028	0.154
Absent	71	37 (52.1)	34 (47.9)		
Present	79	32 (40.5)	47 (59.5)		
SCC antigen level (ng/mL)				3.671	0.055
≤1.5	99	40 (40.4)	59 (59.6)		
>1.5	51	29 (56.9)	22 (43.1)		
Lymphovascular space invasion				0.115	0.734
Absent	134	61 (45.5)	73 (54.5)		
Present	16	8 (50.0)	8 (50.0)		

*SCC* squamous cell carcinoma

* The *P* value was obtained with the Fisher exact test

### PTK6 mRNA and protein expression levels in early-stage cervical squamous cell cancer tissues

**Fig. 1:**
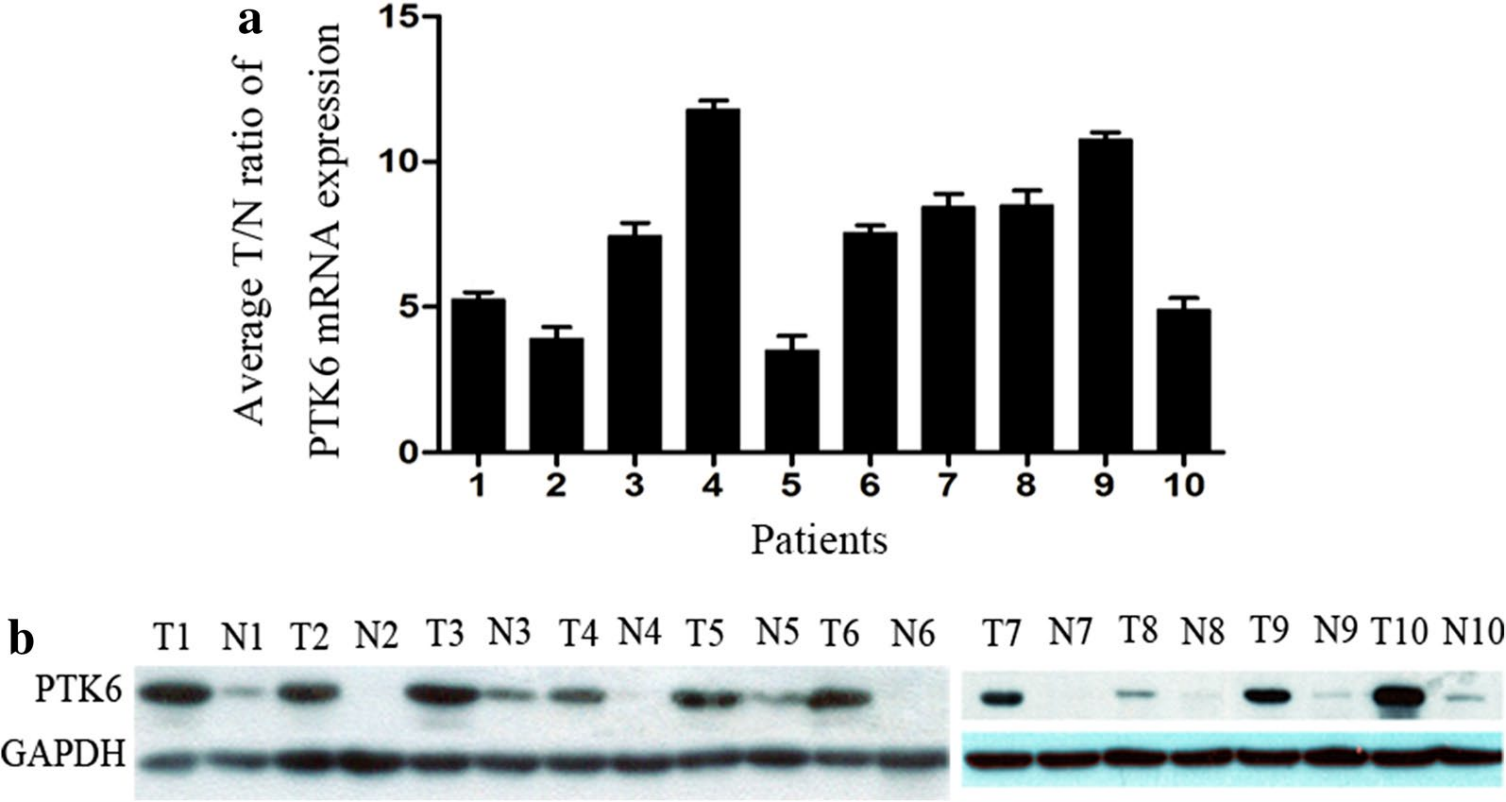
Quantitative reverse transcription-polymerase chain reaction (qRT-PCR) and western blotting analysis of protein tyrosine kinase 6 (PTK6) expression in early-stage cervical squamous cell cancer tissues. **a** qRT-PCR analysis of PTK6 mRNA expression in early-stage cervical squamous cell cancer tissues. The mRNA level of PTK6 in cancer tissues (T) is higher than that in paired adjacent non-tumorous cervical tissues (N), and the range of fold increase (average T/N ratio) was 3.6–11.9. **b** Western blotting analysis of PTK6 protein expression in early-stage cervical squamous cell cancer tissues. The protein level of PTK6 in 10 cervical squamous cell cancer tissues (T) was higher than that in paired adjacent non-tumorous cervical tissues (N). Glyceraldehyde-3-phosphate dehydrogenase (GAPDH) was used as the loading control

qRT-PCR results showed that the mRNA level of PTK6 in cancer tissues was higher than that in adjacent non-tumorous cervical tissues, and the range of fold increase was 3.6–11.9 (Fig. 1a). Similarly, Western blotting showed that the cancer tissues had higher levels of PTK6 protein expression compared with matched non-tumorous tissues (Fig. 1b).

### PTK6 protein expression in cervical cancer tissues and normal cervical epithelia

**Fig. 2:**
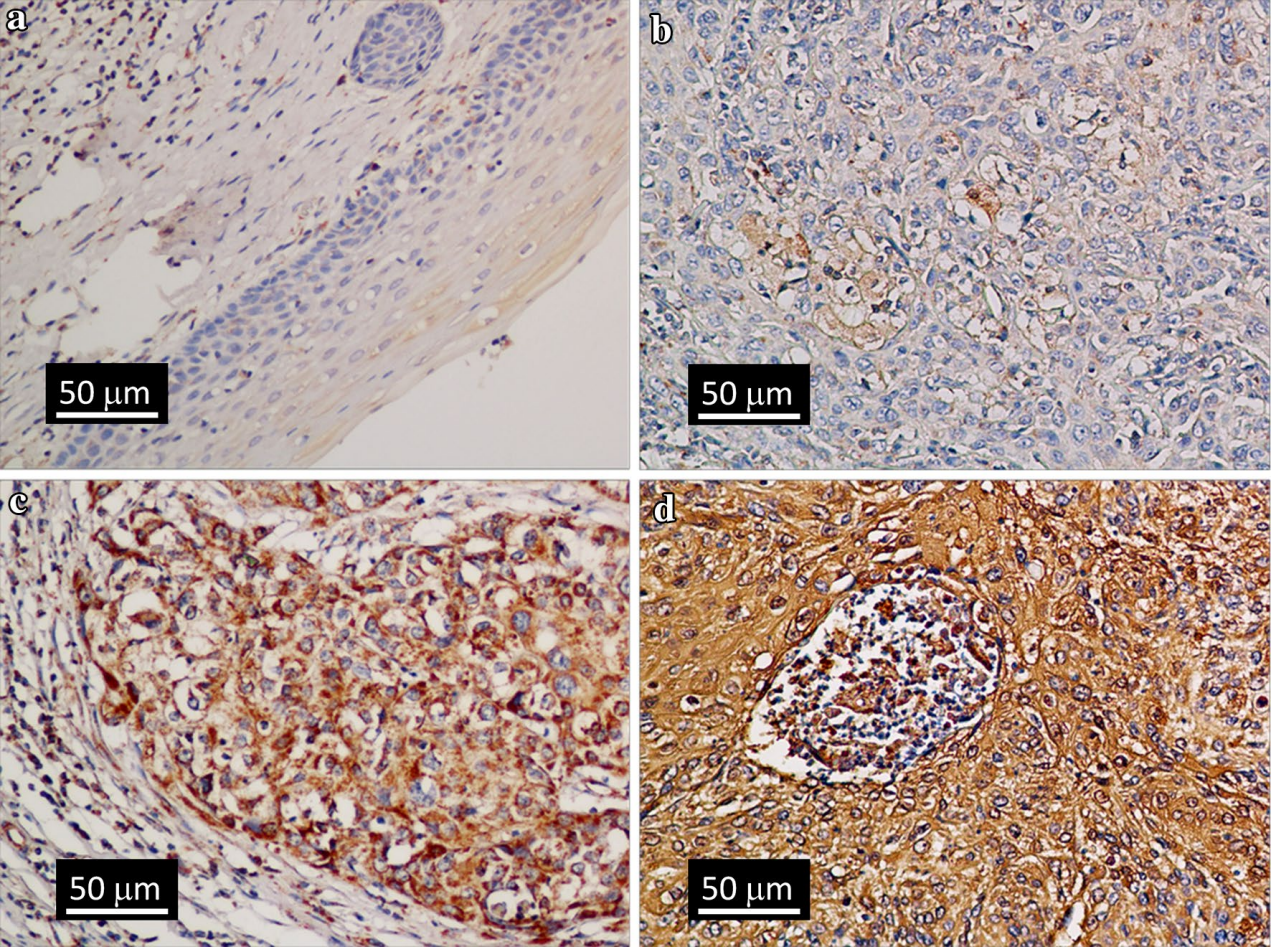
Immunohistochemical analysis of PTK6 expression in normal cervical epithelial tissues and cervical squamous cell cancer tissues. **a** PTK6 protein is not expressed in normal cervical epithelia. **b** PTK6 protein is weakly stained in the cytoplasm of cervical squamous cell cancer; **c** PTK6 protein is moderately stained in the cytoplasm of cervical squamous cell cancer; **d** PTK6 protein is strongly stained in the cytoplasm of cervical squamous cell cancer

PTK6 expression levels were detected with IHC staining in 150 cervical cancer specimens and 10 normal cervical epithelia specimens (Fig. 2). Of the 150 patients, 69 (46.0%) had high cytoplasmic PTK6 expression levels, and 81 (54.0%) showed low cytoplasmic expression levels. No PTK6 protein expression was detected in the 10 normal cervical epithelia samples.

### Relationship between PTK6 expression and clinicopathologic characteristics of patients with early-stage cervical squamous cell cancer

PTK6 expression was associated with tumor grade in patients with early-stage cervical squamous cell cancer (*P* = 0.020); no significant association was detected between PTK6 expression level and age, tumor size, FIGO stage, deep cervical stromal invasion, pelvic lymph node metastasis, SCC antigen level, and lymphovascular space invasion (all *P* > 0.05) (Table 1).

### Association of PTK6 expression and patient survival

**Fig. 3:**
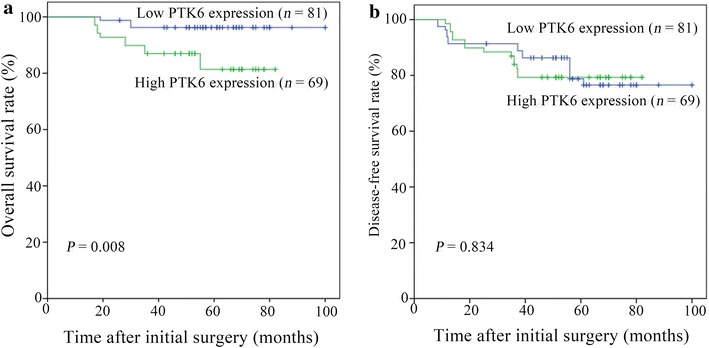
Kaplan-Meier survival curves for early-stage cervical squamous cell cancer patients with low and high PTK6 expression. **a** high PTK6 expression levels were associated with a short OS in patients with early-stage cervical squamous cell cancer (*P* = 0.008); **b** the 5-year DFS rate was similar between these two groups (*P* = 0.834)

The 5-year OS rate of the 150 patients was 90.7%. The 5-year OS rate was significantly lower in patients with high PTK6 expression than in those with low PTK6 expression (81.3% vs. 96.2%, *P* = 0.008) (Fig. 3a); the 5-year DFS rate was similar between these two groups (79.7% vs. 81.5%, *P* = 0.834) (Fig. 3b).

Univariate Cox proportional hazard regression analysis indicated that FIGO stage (*P* = 0.002), pelvic lymph node metastasis (*P* = 0.019), deep cervical stromal invasion (*P* = 0.028), lymphovascular space invasion (*P* < 0.001), and PTK6 expression (*P* = 0.017) were significantly associated with OS of patients with early-stage cervical squamous cell cancer. Multivariate Cox proportional hazard regression analysis revealed that PTK6 expression (*P* = 0.007), FIGO stage (*P* = 0.001), and lymphovascular space invasion (*P* < 0.001) were independent significant prognostic factors for OS (Table 2).

**Table 2:** Univariate and multivariate Cox regression analyses of clinicopathologic variables of patients with early-stage cervical squamous cell cancer for overall survival

Variable	Univariate analysis			Multivariate analysis		
	HR	95% CI	*P* value	HR	95% CI	*P* value
Age (>45 vs. ≤45 years)	2.906	0.911–9.268	0.071			
Tumor size (>4 vs. ≤4 cm)	0.608	0.239–1.545	0.295			
Tumor grade (1 + 2 vs. 3)	1.239	0.446–3.441	0.681			
SCC antigen level (>1.5 vs. ≤1.5 ng/mL)	1.477	0.512–4.264	0.471			
FIGO stage (IB1 + IB2 + IIA1 vs. IIB)	2.111	1.313–3.392	0.002	2.876	1.582–5.229	0.001
Pelvic lymph node metastasis (present vs. absent)	3.569	1.237–10.294	0.019	0.291	0.047–1.789	0.183
Deep cervical stromal invasion (present vs. absent)	5.385	1.205–24.065	0.028	1.548	0.266–8.999	0.626
Lymphovascular space invasion (present vs. absent)	13.212	4.575–38.156	<0.001	19.284	5.958–62.419	<0.001
PTK6 expression (high vs. low)	4.763	1.326–17.112	0.017	5.999	1.622–22.191	0.007

*HR* hazard ratio, *CI* confidence interval, *SCC* squamous cell carcinoma

## Discussion

In our study, we detected PTK6 expression in early-stage cervical squamous cell cancer and analyzed the relationship between PTK6 expression and patients’ clinicopathologic characteristics. Our data indicated that both PTK6 mRNA and protein levels were elevated in cervical squamous cell cancer and the elevated cytoplasmic expression of PTK6 was associated with tumor grade and short patient survival. No PTK6 expression was observed in normal cervical epithelia. Furthermore, our results suggest that the PTK6 expression is an unfavorable independent prognostic factor in patients with early-stage cervical squamous cell cancer.

The function of PTK6 in normal epithelia and in cancer is not fully understood. In normal epithelia, PTK6 is involved in cell differentiation, apoptosis, migration, and tissue repair. In cancer, the role of PTK6 remains controversial. Some studies indicated that PTK6 may act as a tumor suppressor gene [12–14]. Liu et al. [13] found that PTK6 expression was lower in laryngeal squamous cell carcinoma tissues than in adjacent non-tumorous laryngeal epithelial tissues, as measured with Western blotting and RT-PCR. In patients with laryngeal squamous cell carcinoma, low expression of PTK6, as detected by IHC, was associated with short OS and DFS. Ma et al. [14] demonstrated that PTK6 expression was significantly reduced in esophageal squamous cell carcinoma tissues and cell lines compared with non-tumorous tissues or immortalized normal esophageal cell lines. Overexpression of PTK6 in these cells reduced their proliferation in culture and tumor formation in mice.

However, most studies showed that PTK6 was a potential oncogene and played a role in tumorigenesis and tumor development. PTK6 was found to be overexpressed in breast cancer [8], non-small cell lung cancer [9], and ovarian cancer [10]. In vitro studies showed that PTK6 promoted tumor cell migration, invasion, and proliferation [14, 15]. The underlying mechanism by which PTK6 works in cancer biology is gradually being revealed [15–21]. Park et al. [22] demonstrated that PTK6 was involved in tumor cell apoptosis. Down-regulation of PTK6 induced apoptosis in lapatinib-resistant, Her2-positive breast cancer cells by enhancing Bim expression [22]. Harvey et al. [16] demonstrated that PTK6 protected breast cancer cells from autophagic cell death induced by loss of adhesion. In our study, PTK6 was overexpressed in freshly frozen cervical squamous cell cancer specimens, and PTK6 protein was overexpressed in tumor tissues from 150 patients with cervical cancer. Thus, data from the literature and our study suggest that *PTK6* may act as an oncogene in tumor initiation and progression.

The prognostic value of PTK6 in human malignancies was controversial. Studies on esophageal squamous cell carcinoma [12] and laryngeal squamous cell carcinoma [13] showed that low PTK6 expression was significantly associated with low 5-year OS rates for patients. In contrast, studies in other types of cancer, including breast cancer [23] and non-small cell lung cancer [24], showed that high PTK6 expression was significantly associated with low 5-year OS rates. Our study showed that PTK6 overexpression was associated with short survival for patients with cervical cancer. We found that the 5-year OS rate of patients with high PTK6 expression was significantly lower than that of patients with low PTK6 expression (81.3% vs. 96.2%). Furthermore, the Cox proportional hazard regression model revealed that PTK6 expression was an independent prognostic factor for OS. Thus, our data suggest that PTK6 may be used as a prognostic factor for cervical cancer.

The function of PTK6 appears to be highly context- and cell type-specific. In normal epithelial cells, nuclear PTK6 expression is related to the regulation of cell growth, whereas cytoplasmic PTK6 expression may promote tumorigenesis by activating oncogenic signaling pathways in tumor cells [17, 25]. Intracellular localization of PTK6 may determine the outcomes of the PTK6 signaling [26]. In the SW620 colorectal adenocarcinoma cell line, targeting nuclear PTK6 negatively regulated endogenous β-catenin/T cell factor (TCF) transcriptional activity, whereas targeting membrane PTK6 enhanced β-catenin/TCF-regulated transcription [26]. In different cancers, the intracellular location of PTK6 may be different [26]. PTK6 expression was observed on the plasma membrane in human breast tumors and in cell nuclei in well-differentiated prostate tumors [25]. Different types of cross-talk between signaling pathways and PTK6 may exist, which lead to a different PTK6 function. Thus, further studies are needed for a better understanding of the role of PTK6 in cancer biology.

In conclusion, we demonstrated that PTK6 was overexpressed in cervical squamous cell cancer and that high PTK6 expression was associated with short OS. PTK6 expression may be an independent prognostic predictor of cervical cancer.
